# Spontaneous emergence of rudimentary music detectors in deep neural networks

**DOI:** 10.1038/s41467-023-44516-0

**Published:** 2024-01-02

**Authors:** Gwangsu Kim, Dong-Kyum Kim, Hawoong Jeong

**Affiliations:** 1 Department of Physics, Korea Advanced Institute of Science and Technology, Daejeon, 34141 Korea; 2 Center for Complex Systems, Korea Advanced Institute of Science and Technology, Daejeon, 34141 Korea

**Keywords:** Neuroscience, Computational neuroscience, Cognitive neuroscience

## Abstract

Music exists in almost every society, has universal acoustic features, and is processed by distinct neural circuits in humans even with no experience of musical training. However, it remains unclear how these innate characteristics emerge and what functions they serve. Here, using an artificial deep neural network that models the auditory information processing of the brain, we show that units tuned to music can spontaneously emerge by learning natural sound detection, even without learning music. The music-selective units encoded the temporal structure of music in multiple timescales, following the population-level response characteristics observed in the brain. We found that the process of generalization is critical for the emergence of music-selectivity and that music-selectivity can work as a functional basis for the generalization of natural sound, thereby elucidating its origin. These findings suggest that evolutionary adaptation to process natural sounds can provide an initial blueprint for our sense of music.

## Abstract (web summary)

Music is processed by dedicated circuits in the brain. Here, the authors show that these circuits can spontaneously emerge in neural networks by learning natural sound processing, even without explicit training with music.

## Introduction

Music is a cultural universal of all human beings, having common elements found worldwide^1,2^, but it is unclear how such universality arises. As the perception and production of music stem from the ability of our brain to process the information about musical elements^3–7^, the universality question is closely related to how neural circuits for processing music develop, and how universals arise during the developmental process regardless of the diversification of neural circuits derived by the spectacular variety of sensory inputs from different cultures and societies.

In our brain, music is processed by music-selective neural populations in distinct regions of the non-primary auditory cortex; these neurons respond selectively to music and not speech or other environmental sounds^6,8,9^. Several experimental observations suggest that music-selectivity and an ability to process the basic features of music develop spontaneously, without special need for an explicit musical training^10^. For example, a recent neuroimaging study showed that music-selective neural populations exist in not only individuals who had explicit musical training but also in individuals who had almost no explicit musical training^11^. In addition, it was reported that even infants have an ability to perceive multiple acoustic features of music^12,13^, such as melody that is invariant to shifts in pitch level and tempo, similar to adults. One intuitive explanation is that passive exposure to life-long music may initialize the music-selective neural populations^11^, as hearing occurs even during pre-natal periods^14^. However, the basic machinery of music processing, such as harmonicity-based sound segregation, has been observed not only in Westerners but also in native Amazonians who had limited exposure to concurrent pitches in music^15^. These findings raise speculations on whether exposure to music is necessary for the development of an early form of music-selectivity, although subsequent experience-dependent plasticity could further refine the circuits.

Recent modeling studies using artificial deep neural networks (DNNs) have provided insights into the principles underlying the development of the sensory functions in the brain^16–19^. In particular, it was suggested that a brain-like functional encoding of sensory inputs can arise as a by-product of optimization to process natural stimuli in DNNs. For example, responses of DNN models trained for classifying natural images were able to replicate visual cortical responses and could be exploited to control the response of real neurons beyond the naturally-occurring level^20–22^. Even high-level cognitive functions have been observed in networks trained to classify natural images, namely the Gestalt closure effect^23^ and the ability to estimate the number of visual items in a visual scene^24,25^. Furthermore, a DNN trained for classifying music genres and words was shown to replicate human auditory cortical responses^26^, implying that such task-optimization provides a plausible means for modeling the functions of the auditory cortex.

Here, we investigate a scenario in which music-selectivity can arise as a by-product of adaptation to natural sound processing in neural circuits^27–30^, such that the statistical patterns of natural sounds may constrain the innate basis of music in our brain. We show that in a DNN trained for natural sound detection, music is distinctly represented even when music is not included in the training data. We found that such distinction arises from the response of the music-selective units in the feature extraction layer. The music-selective units are sensitive to the temporal structure of music, as observed in the music-selective neural populations in the brain. Further investigation suggests that music-selectivity can work as a functional basis for the generalization of natural sound, revealing how it can emerge without learning music. All together, these results support the possibility that evolutionary pressure to process natural sound contributed to the emergence of a universal template of music.

## Results

### Distinct representation of music in a network trained for natural sound detection including music

We initially tested whether a distinct representation of music can arise in a DNN trained for detecting natural sounds (including music) using the AudioSet dataset^31^. Previous work suggested that a DNN trained to classify music genres and word categories can explain the responses of the music-selective neural populations in the brain^26^. Thus, it was expected that DNNs can learn general features of music to distinguish them from diverse natural sound categories.

**Fig. 1:**
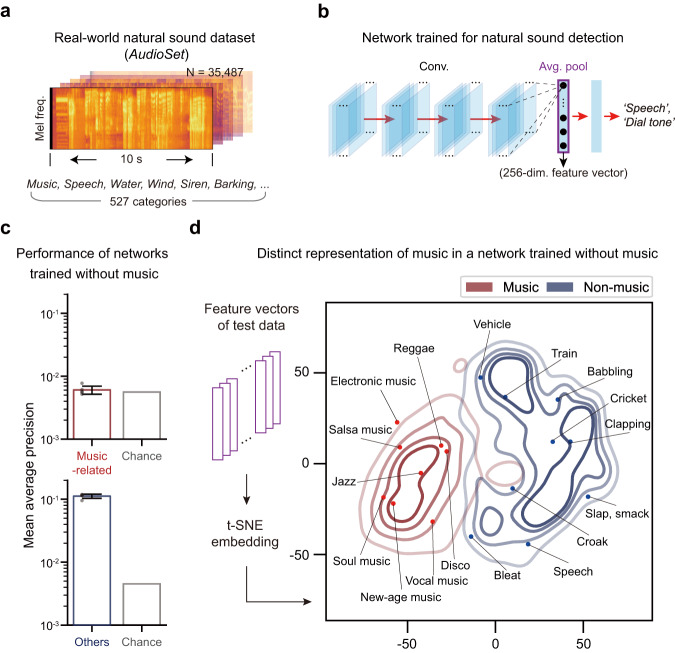
Distinct representation of music in deep neural networks trained for natural sound detection without music. **a** Example log-Mel spectrograms of the natural sound data in the AudioSet^31^. **b** Architecture of the deep neural network used to detect the natural sound categories in the input data. The purple box indicates the average pooling layer. **c** Performance (mean average precision, mAP) of the network trained without music for music-related categories (top, red bars) and other categories (bottom, blue). *n* = 5 independent networks. Error bars represent mean +/− SD. **d** Density plot of the t-SNE embedding of feature vectors obtained from the network in C. The lines represent iso-proportion lines at 80%, 60%, 40%, and 20% levels. Source data are provided as a Source Data file.

The dataset we used consists of 10 s real-world audio excerpts from YouTube videos that have been human-labeled with 527 categories of natural sounds (Fig. 1a, 17,902 training data and 17,585 test data with balanced numbers for each category to avoid overfitting for a specific class). The design of the network model (Fig. 1b and Table S1) is based on conventional convolutional neural networks^32^, which have been employed to successfully model both audio event detection^33^ and information processing of the human auditory cortex^26^. The network was trained to detect all audio categories in each 10 s excerpt (e.g., music, speech, dog barking, etc.). As a result, the network achieved reasonable performance in audio event detection as shown in Fig. S1a. After training, 17,585 test data was presented to the network and the responses of the units in the average pooling layer were used as feature vectors representing the data.

By analyzing the feature vectors of music and non-music data, we show that the network trained with music has a unique representation for music, distinct from other sounds. We used t-distributed stochastic neighbor embedding (t-SNE) to visualize the 256-dimensional feature vectors in two dimensions, which ensures that data close in the original dimensions remain close in two dimensions^34^. The resulting t-SNE embedding shows that the distribution of music data is clustered in a distinct territory of the embedding space, clearly separated from non-music data (Fig. S1b). Such a result is expected; as music was included in the training data, the network can learn the features of music that distinguish music from other categories. Given this, one might expect that such a distinct representation of music would not appear if music were discarded from the training dataset.

### Distinct representation of music in a network trained without music

However, further investigation showed that the distinct representation for music can still arise in a DNN trained without music. To test this, we discarded the data that contain any music-related categories from the training dataset and trained the network to detect other audio events except the music-related categories. As a result, the network was not able to detect music-related categories, but still achieved reasonable performance in other audio event detection (Fig. 1c). Interestingly though, the distribution of music was still clustered in a distinct regime of the t-SNE embedding space, despite the network not being trained with music (Fig. 1d and Fig. S2). This suggests a scenario in which training with music is not necessary for the distinct representation of music by the DNN.

Such observation raises a question on how such distinct representations emerge without training music. Based on previous notions^27–30^, we speculated that features important for processing music can spontaneously emerge as a by-product of learning natural sound processing in DNNs. To rule out other possibilities first, we tested two alternative scenarios: (1) music and non-music can be separated in the representation space of the log-Mel spectrogram using linear features, so that a nonlinear feature extraction process is not required, and (2) units in the network selectively respond to the trained categories but not to unseen categories, so that the distinct representation emerges without any music-related features in the network.

We first found that the distinct representation did not appear when conventional linear models were used. To test this, feature vectors were obtained from data in the log-Mel spectrogram space by applying two conventional models for linear feature extraction: principal component analysis (PCA, Fig. S3a) and a spectro-temporal two-dimensional-Gabor filter bank (GBFB) model of auditory cortical response^35,36^ (Fig. S3c, Methods). Next, we applied the t-SNE embedding method to the obtained vectors, as in Fig. 1d, and analyzed the distribution. Visual inspection suggested that the resulting embedding generated by the PCA and GBFB methods did not show a clear separation between music and non-music (Fig. S3b, d).

To further validate this tendency while avoiding any distortion of data distribution that might arise from the dimension reduction process, we fitted a linear regression model to classify music and non-music in the training dataset by using their feature vectors as predictors and tested the classification performance using the test dataset (Fig. S4a). As a result, the network trained with natural sounds yielded significantly higher accuracy (mAP of the network trained without music: 0.887 ± 0.005, chance level: 0.266) than PCA or GBFB (one-tailed, one-sample Wilcoxon signed-rank test, PCA: mAP = 0.437, U statistic (U) = 15, *p* = 0.031, common language effect size (ES) = 1; GBFB: mAP = 0.828, U = 15, *p* = 0.031, ES = 1, *n* = 5 independent networks). Moreover, the classification accuracy was almost unchanged even when the linear features were used together with the features from the network (Net + PCA: mAP = 0.887 ± 0.004, Net + GBFB: mAP = 0.894 ± 0.004).

**Fig. 2:**
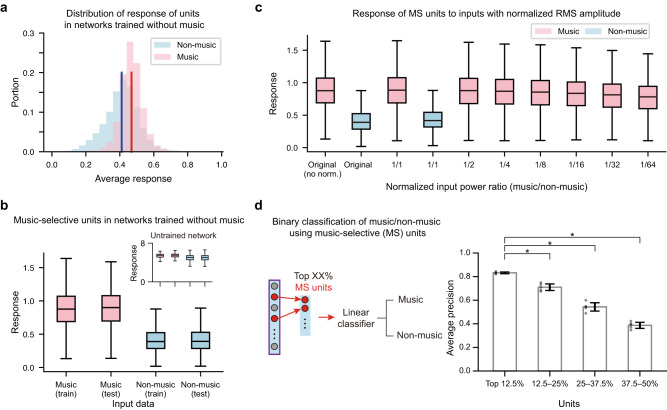
Selective response of units in the network to music. **a** Histograms of the average response of the units for music (red) and non-music (blue) stimuli in networks trained without music. The lines represent the response averaged over all units. **b** Response of the music-selective units to music (red) and non-music stimuli. Inset: Response of the units in the untrained network with the top 12.5% MSI values to music and non-music stimuli. The box represents the lower and upper quartile. The whiskers represent the lower (upper) quartile – (+) 1.5 × interquartile range. n_music, train_ = 4539, n_music, test_ = 3999, n_non-music, train_ = 10,483, and n_non-music_test_ = 11,010 independent sounds. **c** Invariance of the music-selectivity to changes in sound amplitude. Response of the music-selective units to music (red) and non-music (blue) using the training dataset with normalized amplitude. The whiskers represent the lower (upper) quartile – (+) 1.5 × interquartile range. n_music_ = 4539, n_non-music_ = 10,483 independent sounds. **d** Illustration of the binary classification of music and non-music using the response of the music-selective units (left), and the performance of the linear classifier (right). One-tailed Wilcoxon rank-sum test, from left asterisks, U_12.5-25%_ = 25, U_25-37.5%_ = 25, U_37.5-50%_ = 25, p_12.5-25%_ = 0.006, p_25-37.5%_ = 0.006, p_37.5-50%_ = 0.006, ES_12.5-25%_ = 1, ES_25-37.5%_ = 1, ES_37.5-50%_ = 1, *n* = 5 independent networks. Error bars represent mean +/− SD. The asterisks represent statistical significance (*p* < 0.05). Source data are provided as a Source Data file.

Next, we tested whether the distinct representation is due to the specificity of the unit response to the trained categories^37,38^. It is possible that all features learned by the network are specifically fitted to the trained sound categories, so that the sounds of the trained categories would elicit a reliable response from the units while the sounds of unseen categories (including music) would not. To test this, we checked whether the average response of the units to music is significantly smaller than the non-music stimuli. Interestingly, the average response to music was stronger than the average response to non-music (Fig. 2a, one-tailed Wilcoxon rank-sum test, U = 30,340,954, *p* = 4.891 × 10^−276^, ES = 0.689, n_music_ = 3999, n_non-music_ = 11,010). This suggests that features optimized to detect natural sound can also be rich repertoires of music; i.e., the network may have learned features of music throughout the training process even though music was completely absent in the training data.

### Music-selective units in the deep neural network

Based on the above results, we investigated whether units in the network exhibit music-selective responses. We used two criteria to test this: (1) whether some units show a significantly stronger response to music than other sounds, and (2) whether those units encode the temporal structure of music in multiple timescales.

First, we found that some units in the network respond selectively to music rather than other sounds. To evaluate this, we define and quantify the music-selectivity index (MSI) of each network unit as the difference between the average response to music and non-music in the test dataset normalized by their unpooled variance^39^ (i.e., *t*-statistics, Methods). Next, using the train dataset that is independent from the test dataset used to identify MSI, we found that units with the top 12.5% MSI values have an average 2.07 times stronger response to music than to other sounds (Fig. 2b). Thus, these units were considered as putative music-selective units. We found that the same conclusion can be obtained even when the root mean square (RMS) amplitude of all sound inputs was normalized equally, or when the RMS amplitude of music categories was significantly smaller than non-music categories (Fig. 2c, Methods). This suggests that the current results are robust to changes in low-level sound properties such as amplitude. We found that the response of these music-selective units can be exploited for the replication of the basic music classification behavior (Fig. 2d, accuracy: AP: 0.832 ± 0.007) using a linear classifier, for all 25 music genres included in the dataset (Fig. S6). In contrast, using other units with intermediate MSI values showed significantly lower performance, suggesting that the music-selective units provide useful information for processing music.

**Fig. 3:**
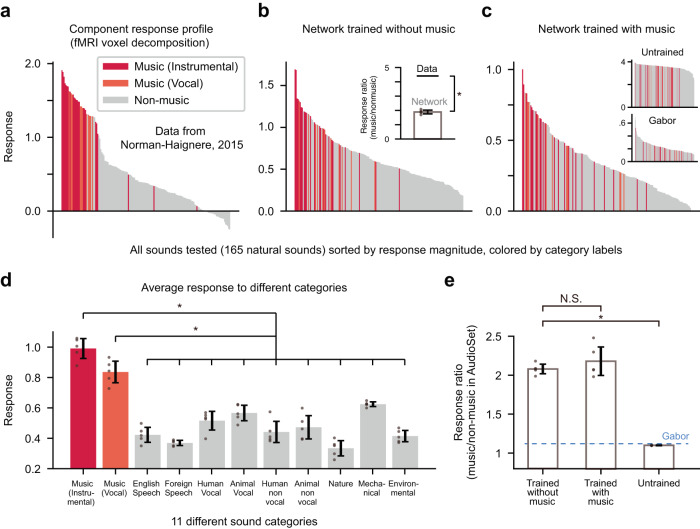
Significance of the music-selectivity emerging in the network trained without music. **a** The component response profile inferred by a voxel decomposition method from human fMRI data (data from Fig. 2d of Norman-Haignere, 2015). Bars represent the response magnitude of the music component to 165 natural sounds. Sounds are sorted in descending order of response. **b** Analysis of the average response of units (in the networks trained without music) with the top 12.5% MSI values (identified with the AudioSet dataset) to the 165 natural sounds data. Inset represents music/non-music response ratio for the fMRI data in (**a**) and the networks trained without music. One-tailed, one-sample Wilcoxon signed-rank test, U = 15, *p* = 0.031, ES = 1, *n* = 5 independent networks. Error bars represent mean +/− SD. **c** The same analysis for the network trained with music, (inset) the randomly initialized network, and the Gabor filter bank model. **d** The average response of music-selective units to each of the 11 sound categories defined in Norman-Haignere, 2015 in the networks trained without music. The music-selective units showed higher responses to the music categories compared to all other non-music sound categories (1-to-1 comparison). One-tailed Wilcoxon singed-rank test, for all pairs, U = 15, *p* = 0.031, ES = 1, *n* = 5 independent networks. **e** The average music/non-music response ratio (Sounds in the training AudioSet dataset) of units with top 12.5% MSI values in each model. Two-tailed Wilcoxon rank-sum test, vs Trained with music: U = 11, *p* = 0.417, ES = 0.44; vs Untrained: U = 0, *p* = 0.006, ES = 0, *n* = 5 independent networks. The asterisks represent statistical significance (*p* < 0.05). Error bars represent mean +/− SD. Source data are provided as a Source Data file.

A previous study showed that the fMRI voxel responses to diverse natural sounds (165 natural sounds) can be decomposed into 6 major components and one of these components exhibits a music-selective response profile^6^ (Fig. 3a). Based on this result, we further examined our model by analyzing the response of music-selective units to the same sound stimuli that were used in the previous human fMRI experiment.

We found that the music-selective network units in the networks trained without music have a higher response to music sounds compared to other non-music sounds in the 165 natural sounds dataset (Fig. 3b, the responses to individual sounds are sorted in descending order). We also noted that this tendency is similarly observed in the networks trained with music, but not in the randomly initialized networks or the Gabor filter bank model (Fig. 3c). To further validate this tendency, we compared the average response of music-selective units in the networks trained without music to each of the 11 sound categories of the 165 natural sounds dataset. As shown in Fig. 3d, the music-selective units consistently showed higher responses to the music categories (both instrumental and vocal) compared to all the other non-music sound categories. This shows that the current model works robustly even on a completely new dataset. Nonetheless, compared to that of the brain, the music-selective units in the network showed a relatively high response to non-music sounds (Fig. 3b inset), which is expected as the network had no refinement process to distinguish music directly. Thus, further refinement of music-selectivity into a more ‘music-exclusive form’ could be expected throughout one’s music-specific experience (at least via a simple linear combination of the existing sub-features) to achieve selectivity at the mature human level.

Comparing the degree of music-selectivity in different models further supported the significance of the music-selectivity emerging in the network trained without music. Figure 3e shows the average response ratio of music-selective units to music and non-music sounds in the training data of AudioSet in different models. The networks trained without music and the networks trained with music showed no statistically significant difference in the degree of music-selectivity (two-tailed Wilcoxon rank-sum test, U = 11, *p* = 0.417, ES = 0.440), and this was significantly greater than that observed in randomly initialized networks or Gabor filter models (one-tailed Wilcoxon rank-sum test, U = 25, *p* = 0.006, ES = 1). We also found that a similar tendency appears when the networks are tested with the 165 natural sounds dataset (music/non-music ratio, trained without music: 1.88 ± 0.13, trained with music: 1.61 ± 0.16, randomly initialized: 1.03 ± 0.0044, Gabor: 0.95); the networks trained without music showed a higher degree of music-selectivity than the other models (one-tailed Wilcoxon rank-sum test, networks trained with music: U = 22, *p* = 0.030, ES = 0.880, randomly initialized networks: U = 25, *p* = 0.006, ES = 1, Gabor filter models: U = 25, *p* = 0.006, ES = 1). Nonetheless, in the case of testing with the 165 natural sounds dataset, the response of the networks trained with the AudioSet could be biased due to the short length of each sound excerpt and relatively small data space (e.g., the 165 natural sounds dataset contains a total of 2 s x 35 music sounds = 70 s of music data).

**Fig. 4:**
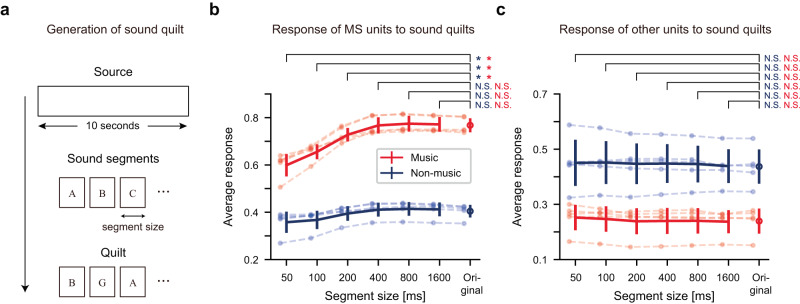
Encoding of the temporal structure of music by music-selective units in the network. **a** Schematic diagram of the generation of sound quilts. A change in the order of the alphabets represents the segment reordering process. **b** Response of the music-selective units to sound quilts made of music (red) and non-music (blue). One-tailed Wilcoxon signed-rank test was used to test whether the response was reduced compared to the original condition. For the music quilts: U_50_ = 15, U_100_ = 15, U_200_ = 15, U_400_ = 8, U_800_ = 0, U_1,600_ = 1, p_50_ = 0.031, p_100_ = 0.031, p_200_ = 0.031, p_400_ = 0.500, p_800_ = 1.000, p_1,600_ = 0.969, ES_50_ = 1.0, ES_100_ = 1.0, ES_200_ = 1.0, ES_400_ = 0.533, ES_800_ = 0, ES_1,600_ = 0.067; for the non-music quilts: U_50_ = 15, U_100_ = 15, U_200_ = 15, U_400_ = 0, U_800_ = 0, U_1,600_ = 0, p_50_ = 0.031, p_100_ = 0.031, p_200_ = 0.031, p_400_ = 1.000, p_800_ = 1.000, p_1,600_ = 1.000, ES_50_ = 1, ES_100_ = 1, ES_200_ = 1, ES_400_ = 0, ES_800_ = 0, ES_1,600_ = 0; n = 5 independent networks. Error bars represent mean +/− SD. **c** Response of the other units to sound quilts made of music (red) and non-music (blue). One-tailed Wilcoxon signed-rank test. For the music quilts: U_50_ = 2, U_100_ = 3, U_200_ = 9, U_400_ = 7, U_800_ = 2, U_1,600_ = 9, p_50_ = 0.938, p_100_ = 0.906, p_200_ = 0.406, p_400_ = 0.594, p_800_ = 0.938, p_1,600_ = 0.406, ES_50_ = 0.133, ES_100_ = 0.2, ES_200_ = 0.6, ES_400_ = 0.467, ES_800_ = 0.133, ES_1,600_ = 0.6; for the non-music quilts: U_50_ = 3, U_100_ = 2, U_200_ = 5, U_400_ = 1, U_800_ = 1, U_1,600_ = 4, p_50_ = 0.906, p_100_ = 0.938, p_200_ = 0.781, p_400_ = 0.969, p_800_ = 0.969, p_1,600_ = 0.844, ES_50_ = 0.2, ES_100_ = 0.133, ES_200_ = 0.333, ES_400_ = 0.067, ES_800_ = 0.067, ES_1,600_ = 0.267; *n* = 5 independent networks. Error bars represent mean +/- SD. The asterisks indicate statistical significance (*p* < 0.05). N.S.: non-significant. Source data are provided as a Source Data file.

Second, we found that the music-selective units in the network showed sensitivity to the temporal structure of music, replicating previously observed characteristics of tuned neural populations in the human auditory cortex^6,40,41^. While music is known to have distinct features in both long and short timescales^6,41^, it is possible that the putative music-selective units only encode specific features of music in a specific (especially short) timescale. To test this, we adopted the ‘sound quilting’ method^41^ (Fig. 4a, Methods): the original sound sources were divided into small segments (50–1600 ms in octave range) and then reordered while considering smooth connections between segments. We note that the stimuli used for the generation of the sound quilt (sounds in the training dataset) are independent of the dataset used to identify music-selective units (the test dataset). This shuffling method preserves the acoustic properties of the original sound on a short timescale but destroys it on a long timescale. It has been shown that the response of music-selective neural populations in the human auditory cortex is reduced when the segment size is small (e.g., 30 ms) so that the temporal structure of the original sound is broken^6^. Similarly, after recording the response of the music-selective units to such sound quilts of music, we found that their response is correlated with the segment size (music quilt: Pearson’s *r* = 0.601, *p* = 4.447 × 10^−4^). The response to the original sound was not statistically significantly higher than the response to 800 ms segment inputs, but it was higher than the response to 50 ms segment inputs (Fig. 4b, original: 0.768 ± 0.030; 800 ms: 0.775 ± 0.033; 50 ms: 0.599 ± 0.047; one-tailed Wilcoxon signed-rank test, 800 ms: U = 0, *p* = 1.000, ES = 0, 50 ms: U = 15, *p* = 0.031, ES = 1). In addition, this tendency was consistently observed in sound quilts of various genres (Fig. S7), suggesting that the network units are encoding common features of various music styles.

To test whether or not the effect is due to the quilting process itself, we provided quilts of music to the other non-music-selective units. In this condition, we found that the average response remains constant even when the segment size changes (Fig. 4c). Furthermore, when quilted natural sound inputs were provided, the correlation between the response of the music-selective units and the segment length was weaker than when quilted music inputs were provided (Fig. 4b, non-music quilt: Pearson’s *r* = 0.38, *p* = 0.036), even though the significant correlation was observed for both types of inputs. Notably, all these characteristics of the network trained without music replicate those observed in the human brain^6,41^.

Further analysis showed that the linear features of GBFB methods do not show the properties observed in the DNN model. The filters with the top 12.5% MSI values showed a 1.12 times stronger response to music than to other sounds in the training dataset on average (Fig. 3e and Fig. S4b), which is far lower than that of the network units. Furthermore, the response profile of those top 12.5% spectro-temporal filters to sound quilts (Fig. S4c, same analysis as in Fig. 4b) did not show the patterns observed for the MS units in the network, implying that those features do not encode the temporal structure of music. Similarly, our analysis showed that the units in the random network do not show those properties observed in the network trained without music (Fig. S5). These results further suggest that the conventional spectro-temporal filter or features in randomly-initialized network cannot explain the results that the units in the network encode the features of music.

### Music-selectivity as a generalization of natural sounds

Then how does music-selectivity emerge in a network trained to detect natural sounds even without training music? In the following analysis, we found that music-selectivity can be a critical component to achieve generalization of natural sound in the network, and thus training to detect natural sound spontaneously generates music-selectivity.

**Fig. 5:**
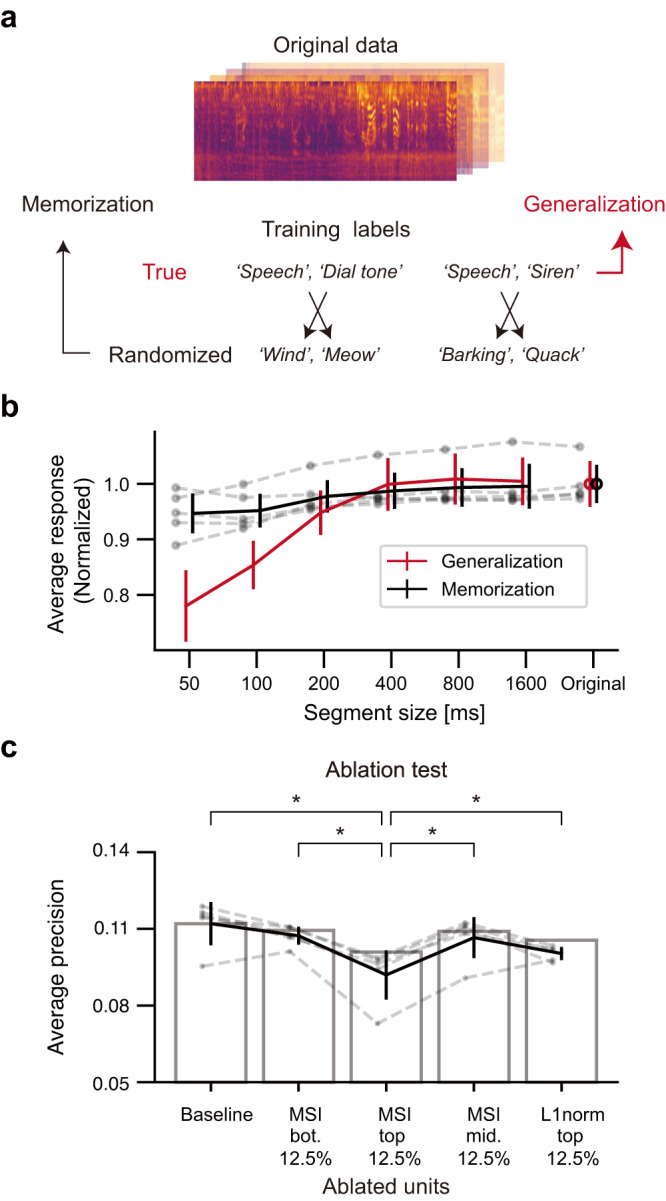
Music-selectivity as a generalization of natural sounds. **a** Illustration of network training to memorize the data by randomizing the labels. **b** Response of the units with the top 12.5% MSI values to music quilts in the networks trained with randomized labels (black, memorization) compared to that of the network in Fig. 4b (red, generalization). To normalize the two conditions, each response was divided by the average response to the original sound from each network. One-tailed Wilcoxon rank-sum test, U_50_ = 25, U_100_ = 25, U_200_ = 17, U_400_ = 14, U_800_ = 10, U_1,600_ = 15, p_50_ = 0.006, p_100_ = 0.006, p_200_ = 0.202, p_400_ = 0.417, p_800_ = 0.735, p_1,600_ = 0.338, ES_50_ = 1, ES_100_ = 1, ES_200_ = 0.68, ES_400_ = 0.56, ES_800_ = 0.4, ES_1,600_ = 0.6, *n* = 5 independent networks. Error bars represent mean +/− SD. **c** Performance of the network after the ablation of specific units. One-tailed Wilcoxon signed-rank test, MSI top 12.5% vs Baseline: U = 15, *p* = 0.031, ES = 1; vs MSI bot. 12.5%: U = 15, *p* = 0.031, ES = 1, vs MSI mid. 12.5%: U = 15, *p* = 0.031, ES = 1, vs L1 norm top 12.5%: U = 15, *p* = 0.031, ES = 1. The asterisks indicate statistical significance (*p* < 0.05). *n* = 5 independent networks. Error bars represent mean +/− SD. Source data are provided as a Source Data file.

Clues were found from the observation that as the task performance of the network increases over the course of training, the distinct representation of music and non-music becomes clearer in the t-SNE space (Fig. S8). Based on this intuition, we hypothesized that music-selectivity can act as a functional basis for the generalization of natural sound, so that the emergence of music-selectivity may directly stem from the ability to process natural sounds. To test this, we investigated whether music-selectivity emerges when the network cannot generalize natural sounds (Fig. 5a). To hinder the generalization, the labels of the training data were randomized to remove any systematic association between the sound sources and their labels, following a previous work^42^. Even in this case, the network achieved high training accuracy (training AP > 0.95) by memorizing all the randomized labels in the training data, but showed a test accuracy at the chance level as expected.

We found that the process of generalization is indeed critical for the emergence of music-selectivity in the network. For the network trained to memorize the randomized labels, the distributions of music and non-music were clustered to some degree in the t-SNE embedding space (Fig. S9). However, more importantly, units in the network trained to memorize did not encode the temporal structure of music. To test this, we analyzed the response of the units with the top 12.5% MSI values in the network trained to memorize using sound quilts of music as in Fig. 4b. We found that even if the segment size of the sound quilt changed, the response of the units remained mostly constant, unlike the music-selective units in the network trained to generalize natural sounds (Fig. 5b). This supports our hypothesis that music-selectivity is based on the process of generalization of natural sounds.

To further investigate the functional association, we performed an ablation test (Fig. 5c), in which the response of the music-selective units is silenced and then the sound event detection performance of the network is evaluated. If the music-selective units provide critical information for the generalization of natural sound, removing their inputs would reduce the performance of the network. Indeed, we found that ablation of the music-selective units significantly deteriorates the performance of the network (Fig. 5c, MSI top 12.5% vs Baseline: U = 1,560,865, *p* = 3.027 × 10^−211^, ES = 0.917, one-tailed Wilcoxon signed-rank test). This effect was weaker when the same number of units with intermediate/bottom MSI values were silenced. Furthermore, the performance drop was even greater than that of ablating the units showing strong responses to inputs on average (MSI top 12.5%-L1norm top 12.5%: U = 1,223,566, p = 9.740 × 10^−60^, ES = 0.719, one-tailed Wilcoxon signed-rank test). This suggests that music and other natural sounds share key features, and thus music-selective units can play a functionally important role not only in music processing but also in natural sound detection.

Finally, we investigated the role of speech in the development of music-selectivity by removing speech sounds from the training dataset. We found that music-selectivity can emerge even without training speech, but speech can play an important role for units to encode the temporal structure of music in long time scales. To investigate the role of speech, data with music-related labels or speech-related labels (all labels under the speech hierarchy) were removed from the training dataset. Then, we trained the network with this dataset and compared the main analysis results with those of the network trained without music.

We found that even if speech data was removed from the training dataset, distinct clustering of music and non-music was still observed in the t-SNE embedding space (Fig. S10a). Likewise, some units in the network still exhibited music-selectivity, showing 1.77 times stronger response to music than non-music on average (Fig. S10b). However, further analysis using sound quilts showed that the response of music-selective units in the network trained without speech shows less sensitivity to the size of the segment compared to that of the network trained with speech (Fig. S10c). This suggests that training with speech helps the network units to acquire long-range temporal features of music.

Nevertheless, the learned features did not contain much information about speech, unlike music. First, our t-SNE analysis showed that some weak degree of clustering of speech can still emerge in the t-SNE space without training speech (Fig. S11a). Next, we checked whether the average response of the units to speech is significantly higher than the non-music stimuli. There was no statistically significant evidence that the average response to speech sounds is stronger than that to non-speech sounds (Fig. S11b). Furthermore, we evaluated the speech-selectivity index (SSI) of each network unit as the difference between the average response to speech and non-speech in the test dataset normalized by their unpooled variance (Fig. S11c, similar to MSI). The units with the top 12.5% SSI values showed a 1.31 times stronger response to speech than other sounds in the training dataset on average, which is far lower than that of using music (Fig. 2b). Furthermore, the response profile of putative speech-selective units (top 12.5% SSI values) to sound quilts of speech did not show the patterns observed for MS units, implying that the network units do not encode the temporal structure of speech^41^ (Fig. S11d). We also tested the encoding for other sound categories including vehicle, animal, and water sounds (categories with the top 3 data numbers in the training dataset, except music and speech) using the network trained without music, but we found that the properties observed for the other sound categories are less significant than that in music (Fig. S12).

## Discussion

Here, we put forward the notion that neural circuits for processing the basic elements of music can develop spontaneously as a by-product of adaptation for natural sound processing.

Our model provides a simple explanation about why a DNN trained to classify musical genres replicated the response characteristics of the human auditory cortex^26^, although it is unlikely that the human auditory system itself has been optimized to process music. This is because training with music would result in learning general features for natural sound processing, as music and natural sound processing share a common functional basis. This explanation is also valid for the observation that the auditory perceptual grouping cue of humans can be predicted from statistical regularities of music corpora^30^.

The existence of a basic ability to perceive music in multiple non-human species is also explained by the model. Our analysis showed that music-selectivity lies on the continuum of learning natural sound processing. If the mechanism also works in the brain, such ability would appear in a variety of species adapted to natural sound processing, but to varying degrees. Consistent with this idea, the processing of basic elements of music has been observed in multiple non-human species: octave generalization in rhesus monkeys^43^, the relative pitch perception of two-tone sequences in ferrets^44^, and a pitch perception of marmoset monkeys similar to that of humans^45^. Neurophysiological observations that neurons in the primate auditory cortex selectively respond to pitch^46^ or harmonicity^47^ were also reported, further supporting the notion. A further question is whether phylogenetic lineage would reflect the ability to process the basic elements of music, as our model predicts that music-selectivity is correlated with the ability to process natural sounds. However, it should be noted that there may be differences between the distribution of the modern sound data used for training (e.g., vehicle, mechanical sounds) and the sound data driving evolutionary pressure. Furthermore, some studies reported a lack of functional organization in different species for processing harmonic tones (in macaques^48^) or music (in ferrets^49^), suggesting that higher-order demands for processing music could play an important role in the development of the mature music-selective functional organizations found in humans.

The scope of the current model is limited to how the innate machinery of music processing can arise initially, and does not account for complete experience-dependent development of musicality. The results here do not preclude the possibility that further experience-dependent plasticity could significantly refine our ability to process music (including the music-selectivity), and indeed, several studies supporting this notion have been reported^48,49^. The current results do not conflict with this notion; future studies that include experience-dependent development would be able to clarify the role of each process.

The current CNN-based model is not a model that fully reflects or mimics the structure or mechanism of the brain. For example, as our CNN models are based on a feedforward connectivity structure, the intracortical connections or top-down connections that exist in the brain cannot be reflected in the models. Furthermore, although the learning of the DNN models is governed by the backpropagation of errors, it is at least unclear how the brain may compute errors with respect to a specific computational objective and how the network assigns credits to the tremendous number of synaptic weights^16^. Nonetheless, despite these discrepancies, previous works have reported that there is a hierarchical correspondence between the layers of task-optimized CNNs and regions of the auditory cortex^26^. Our results thus support the notion that optimizing for a specific computational goal can drive different systems to converge to have a similar representation for processing natural stimuli^21^, suggesting the possible scenario that the development of music-selectivity in the brain may have been guided by a similar computational goal.

Recent studies suggest that music has a common element found worldwide, but there is a considerable variation in musical styles among distinct societies covering multiple geographical regions^1,2^. One interesting but unanswered question is what might create these commonalities or differences in music from various societies. According to the current model, the basic perceptual elements to process music are shaped by the statistics of the auditory environment. Therefore, commonalities in music may have emerged from a common element of the auditory environment, and distinct musical styles may have been affected by the difference in the statistics of the auditory environment (plausibly due to geographical/historical distinction). For example, as speech sounds are common and rich sources in the auditory environment regardless of geographical distinction, speech may have played a key role in the development of common features of music. Indeed, our analysis showed that information on the temporal structure of music in a long time scale can be reduced when speech sounds are removed from the training dataset. We expect that future studies would reveal the relationship between distinct auditory environments and musical styles.

Our results also provide insights into the workings of audio processing in DNNs. Recent works showed that the class selectivity of DNN units is a poor predictor of the importance of the units and can even impair generalization performance^50,51^, possibly because it can induce overfitting to a specific class. On the other hand, we found that music-selective units are important for the natural sound detection task, and a good predictor of DNN performance. One possible explanation is that the music-selective units have universal features for the generalization of other natural sounds rather than specific features for specific classes, and thus removing them hinders the performance of the DNN. Thus, these results also support the notion that the general features of natural sounds learned by DNNs are key features that make up music.

In summary, we demonstrated that music-selectivity can spontaneously arise in a DNN trained with real-world natural sounds without music, and that the music-selectivity provides a functional basis for the generalization of natural sound processing. By replicating the key characteristics of the music-selective neural populations in the brain, our results encourage the possibility that a similar mechanism could occur in the biological brain, as suggested for visual^22–24^ and navigational^52^ functions using task-optimized DNNs. Our findings support the notion that ecological adaptation may initiate various functional tunings in the brain, providing insight into how the universality of music and other innate cognitive functions arises.

## Methods

All simulations were done in Python using the PyTorch and TorchAudio framework.

### Neural network model

Our simulations were performed with conventional convolutional neural networks for audio processing. At the input layer, the original sound waveform (sampling rate = 22,050 Hz) was transformed into a log-Mel spectrogram (64 mel-filter banks in the frequency range of 0 Hz to 8000 Hz, window length: 25 ms, hop length: 12.5 ms). Next, four convolutional layers followed by a batch-normalization layer and a max-pooling layer (with ReLU activation and a dropout rate of 0.2) extracted the features of the input data. The global average pooling layer calculated the average activation of each feature map of the final convolutional layer. These feature values were passed to two successive fully connected layers, and then a sigmoid function was applied to generate the final output of the network. The detailed hyperparameters are given in Table S1.

### Stimulus dataset

The dataset we used is the AudioSet dataset^31^, a collection of human-labeled (multi-label) 10 s clips taken from YouTube videos. We used a balanced dataset (17,902 training data and 17,585 test data from distinct videos) consisting of 527 hierarchically organized audio event categories (e.g., ‘classical music’ under ‘music’). Music-related categories were defined as all classes under the music hierarchy, and some classes under the human voice hierarchy (‘Singing’, ‘Humming’, ‘Yodeling’, ‘Chant’, ‘Male singing’, ‘Female singing’, ‘Child singing’, ‘Synthetic singing’, and ‘Rapping’). To completely remove music from the training dataset, we independently validated the presence of music in the other data in the balanced training dataset and found that about 4.5% (*N* = 507) of the data contains music without a music-related label. Some typical errors were as follows: (1) the music is too short or cut off, (2) the volume of music is lower than other sounds, (3) simple errors of the human-labeler. The names of the excluded erroneous files are documented on our GitHub repository.

Each excerpt in the dataset is intrinsically multi-labeled as different sounds generally co-occur in a natural environment, but a sufficient number of data was selected to contain only music-related categories (3999 in the training set and 4539 in the test set) and no music-related categories (11,010 in the training set and 10,483 in the test set). To test for the distinct representation of music, the data were reclassified into music, non-music, and mixed sound, and then mixed sounds were excluded in the analysis of music-selectivity. This was required because some data that contained music-related categories can also contain other audio categories (e.g., music + barking).

The 165 natural sound dataset and the component response profile data in Fig. 3a was obtained from the following publicly available repository provided by the authors: https://github.com/snormanhaignere/natsound165-neuron2015. The data consists of 11 diverse natural sound categories: ‘Music (instrumental)’, ‘Music (vocal)’, ‘English speech’, ‘Foreign speech’, ‘Human vocal’, ‘Animal vocal’, ‘Human non-vocal’, ‘Animal non-vocal’, ‘Nature, Mechanical’, and ‘Environmental’. The data was resampled to match the sampling rate of the original dataset (22,050 Hz), and the RMS (root mean square) amplitude of each sound was normalized to the average RMS amplitude of the data previously used for network training (AudioSet). Then the response of the music-selective units to the 165 sound inputs was recorded.

### Network training

We trained the network to detect all sound categories in each 10 s clip (multi-label detection task). To that aim, the loss function of the network was chosen as the binary cross-entropy between the target (y) and the output (x), which is defined as

1 $$l=-\left[y\cdot \log x+\left(1-y\right)\cdot \log \left(1-x\right)\right]$$

for each category. For optimizing this loss function, we employed the AdamW optimizer with weight decay = 0.01^53^. Each network was trained for 100 epochs (200 epochs for the randomized labels) with a batch size of 32 and the One Cycle learning rate (LR) method^54^. The One Cycle LR is an LR scheduling method for faster training and preventing the network from overfitting during the training process. This method linearly anneals the LR from the initial LR ${4\times 10}^{-5}$ to the maximum LR 0.001 for 30 epochs and then from the maximum LR to the minimum LR ${4\times 10}^{-9}$ for the remaining epochs. For every training condition, simulations were run for five different random seeds of the network. The network parameters used in the analysis were determined from the epoch that achieved the highest average precision over the training epochs with 10% of the training data used as a validation set.

The previously reported audio event detection performance of a CNN trained with the balanced training dataset is mAP = 0.221^33^ (12 convolutional layers, no data augmentation) and ours is mAP = 0.152 (4 convolutional layers). We considered this as a reasonable difference as the current CNN model has a smaller number of parameters compared to the baseline model (80,753,615 vs 1,278,191), and we did not focus on performance optimization.

### Analysis of the responses of the network units

The responses of the network units in the average pooling layer were analyzed as feature vectors (256 dimensions) representing the data. Following a previous experimental study^39^, the music-selectivity index of each unit was defined as

2 $${{{{{\rm{MSI}}}}}}=\frac{{m}_{{music}}-{m}_{{non}-{music}}}{\sqrt{\frac{{{s}_{{music}}}^{2}}{{n}_{{music}}}+\frac{{{s}_{{non}-{music}}}^{2}}{{n}_{{non}-{music}}}}}$$

where *m* is the average response of a unit to music and non-music stimulus, *s* is the standard deviation, and *n* is the number of each type of data. The units (features) with the top 12.5% MSI values were identified in the same way even in the case of randomly initialized networks or the conventional filter bank model. The MSI of the memorization network were calculated by using the data with the correct labels (not the shuffled label that was used to train the memorization network) in the same way as other networks. Likewise, the response of the units with the top 12.5% MSI in the memorization network was analyzed for the sound quilt analysis in Fig. 5b.

To quantify the degree of music-selectivity in different networks and with different datasets, we calculated the average ratio of the response of music-selective units to music and non-music (as the MSI value is dependent on the number of input data).

### Testing invariance of the music-selectivity to changes in sound amplitude

In Fig. 2c, we further tested the robustness of the music-selectivity to changes in sound amplitude. To control the amplitude of sounds, we first obtained the average root mean square (RMS) amplitude of the entire training data and then normalized all individual test data to have an RMS amplitude equal to this value. Even when we normalize the sound amplitude, the music-selective units in the network (previously identified with the original data) showed a stronger response to music than other non-music sounds at almost the same degree (training dataset: 1.97 times, test dataset: 1.95 times). To further test this robustness, we gradually reduced the amplitude of the music sounds (so that the RMS amplitude of music was smaller than that of non-music, power ratio: 1/2, 1/4, 1/8, 1/16, 1/32, and 1/64, RMS amplitude ratio: square root of the power ratio). As can be seen in the figure, even when the power ratio between music and non-music is 1:64 (i.e., RMS amplitude ratio: 1:8), the network shows a significantly stronger response to music than non-music (training dataset: 1.70 times, test dataset: 1.68 times).

### Extraction of linear features using conventional approaches

The linear features of the log-Mel spectrogram of the natural sound data were extracted by using principal component analysis (PCA) and the spectro-temporal two-dimensional-Gabor filter bank (GBFB) model following previous works^35,36^. In the PCA case, feature vectors were obtained from the top 256 principal components (total explained variance: 0.965). In the case of the GBFB model, a set of Gabor filters were designed to detect specific spectro-temporal modulation patterns, which are defined as

3 $$g\left(k,n\right)={s}_{{w}_{k}}\left(k-{k}_{0}\right)\cdot {s}_{{w}_{n}}\left(n-{n}_{0}\right){\cdot h}_{\frac{{\nu }_{k}}{2{w}_{k}}}\left(k-{k}_{0}\right){\cdot h}_{\frac{{\nu }_{n}}{2{w}_{n}}}\left(n-{n}_{0}\right)$$

4 $${h}_{b}\left(x\right)=\left\{\begin{array}{c}0.5-0.5\cos \left(\frac{2\pi x}{b}\right)\quad-\frac{b}{2} \, < \, x \, < \, \frac{b}{2}\\ 0\hfill{otherwise}\end{array}\right.$$

5 $${s}_{w}\left(x\right)=\exp ({iwx})$$

where *k* and *n* represent the channel and time variables (center: *k*_*0*_ and *n*_*0*_), *w*_*k*_ is the spectral modulation frequency, *w*_*n*_ is the temporal modulation frequency, and *ν* is the number of semi-cycles under the envelope. The distribution of the modulation frequencies was designed to limit the correlation between filters as follows,

6 $${{w}_{x}}^{i+1}={{w}_{x}}^{i}\frac{1+\frac{c}{2}}{1-\frac{c}{2}},\, \qquad c={d}_{x}\frac{8}{{\nu }_{x}}$$

Here, we used *d*_*k*_ = 0.1, *d*_*n*_ = 0.045, *ν*_*k*_ = *ν*_*n*_ = 3.5, with *w*_*k, max*_ = *w*_*n, max*_ = π/4, resulting in 15 spectral modulation frequencies, 18 temporal modulation frequencies, and 263 independent Gabor filters (15 × 18–7). Next, a log-Mel spectrogram was convolved with each Gabor filter and then averaged after applying ReLU nonlinearity to generate the 263-dimensional feature vector representing the data. Nonetheless, our investigation showed that the specific choice of the parameters does not change the results significantly.

### Generation of sound quilts

Sound quilts were created according to the algorithm proposed in a previous work^41^. The balanced training dataset (Fig. 4) was used as the quilting source material. First, the original sound sources were divided into small segments of equal size (50–1600 ms in octave range). Next, these segments were reordered while minimizing the difference between the segment-to-segment change in log-Mel spectrogram of the original sound and that of the shuffled sound. We concatenated these segments while minimizing the boundary artifacts by matching the relative phase between segments at the junction^41^. The quilts were cut to 8 s and zero padding was applied to the remaining 2 s to ensure that there was no difference in length of the sound content between the sound quilts.

### Ablation test

In the ablation test, music-related categories were excluded from the performance measure. The units in the network were grouped based on MSI value: top 12.5% units (MS units, *N* = 16), middle 43.75–56.25% units, and bottom 12.5% units. In addition, we grouped the units that showed a strong average response to the test data (top 12.5% L1 norm). The response of the units in each group was set to zero to investigate their contribution to natural sound processing.

### Statistical analysis

All statistical variables, including the sample sizes, exact *P* values, and statistical methods, are indicated in the corresponding texts or figure legends. The common language effect size was calculated as the probability that a value sampled from one distribution is greater than a value sampled from the other distribution. No statistical method was used to predetermine sample size.

### Reporting summary

Further information on research design is available in the Nature Portfolio Reporting Summary linked to this article.

### Supplementary information

Supplementary Information

Reporting Summary

### Source data

Source Data

## Data Availability

The AudioSet dataset is available at https://research.google.com/audioset/^31^. The human fMRI data^6^ used in Fig. 3 are available at https://github.com/snormanhaignere/natsound165-neuron2015. Source data are provided with this paper.

## Code availability

The python codes used in this work are available at https://zenodo.org/doi/10.5281/zenodo.10081609^55^.
