# Supplementary material for: Spontaneous emergence of rudimentary music detectors in deep neural networks
Source: Nat Commun. 2024 Jan 2;15:148. doi: 10.1038/s41467-023-44516-0 (PMC10761941; doi:10.1038/s41467-023-44516-0)
Supplement: Supplementary file 1 — Supplementary Information [file 41467_2023_44516_MOESM1_ESM.pdf]

Supplementary Information

Spontaneous emergence of rudimentary music detectors in deep neural networks

Gwangsu Kim[1], Dong-Kyum Kim[1], and Hawoong Jeong[1,2*]

[1]Department of Physics, Korea Advanced Institute of Science and Technology, Daejeon 34141, Korea

[2]Center for Complex Systems, Korea Advanced Institute of Science and Technology, Daejeon 34141, Korea

*Corresponding author. Email: hjeong@kaist.edu

Supplementary Information

Supplementary Fig. 1. Distinct representation of music in deep neural networks trained for natural sound detection with music.

Supplementary Fig. 2. Results of t-SNE embedding for various perplexity values.

Supplementary Fig. 3. T-SNE embedding of the feature vectors obtained by linear methods.

Supplementary Fig. 4. Analysis of feature vectors obtained by linear methods.

Supplementary Fig. 5. Analysis of the feature vectors of randomly-initialized networks.

Supplementary Fig. 6. Music classification task using the music-selective units.

Supplementary Fig. 7. Analysis of response of the units in networks trained without music to sound quilts of different music genres.

Supplementary Fig. 8. Density plots of the t-SNE embeddings of music (red) and non-music (blue) over the training epochs.

Supplementary Fig. 9. T-SNE embedding of the feature vectors of the network trained to memorize natural sounds with randomized labels.

Supplementary Fig. 10. Analysis of the feature vectors of networks trained without music or speech.

Supplementary Fig. 11. Representation of speech in networks trained without music or speech.

Supplementary Fig. 12. Analysis of the encoding of other sound categories.

Supplementary Table 1. Summary of the network architecture.

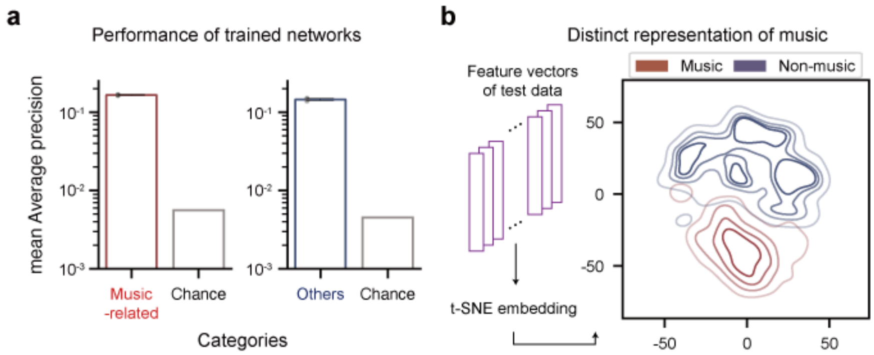

Supplementary Fig. 1. Distinct representation of music in deep neural networks trained for natural sound detection with music.

(**a**) Performance of the trained network for music-related categories (left, red bars) and other categories (right, blue). Error bars represent mean +/- SD. n = 5 independent networks. (**b**) Density plot of the t-SNE embedding of feature vectors obtained from the trained network. The lines represent iso-proportion lines at 80%, 60%, 40%, and 20% levels. Source data are provided as a Source Data file.

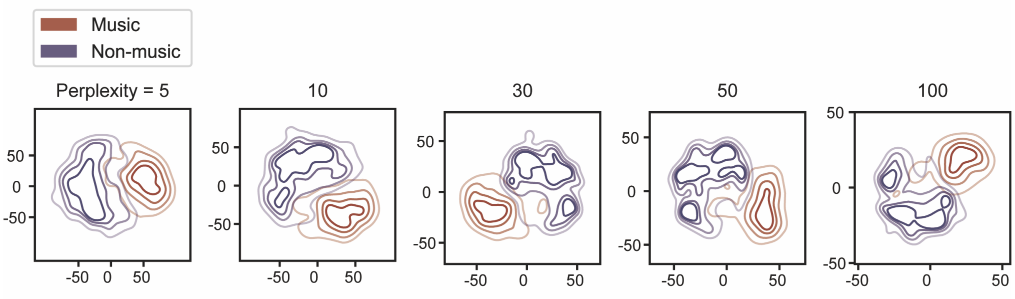

Supplementary Fig. 2. Results of t-SNE embedding for various perplexity values.

Density plot of the t-SNE embedding of feature vectors of network trained without music for perplexity values of 5, 10, 30, 50, and 100. As can be seen in the figure, distinct clustering of music and non-music for the network trained without music was robustly maintained in the embedding space under a 20-fold variation of perplexity. Such robustness suggests that music and non-music are well separated in a high-dimensional feature vector space.

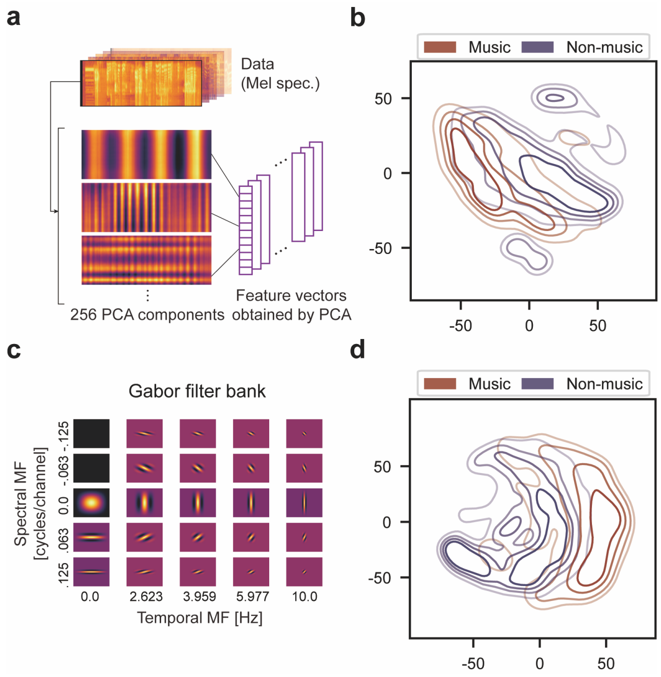

Supplementary Fig. 3. T-SNE embedding of the feature vectors obtained by linear methods.

(**a**) Example PCA components obtained from the data. (**b**) Density plot of the t-SNE embedding of feature vectors obtained from PCA. The lines represent iso-proportion lines at 80%, 60%, 40%, 20% levels. (**c**) Example spectro-temporal Gabor filters. MF: modulation frequency. (**d**) Density plot of the t-SNE embedding of feature vectors obtained from Gabor filters.

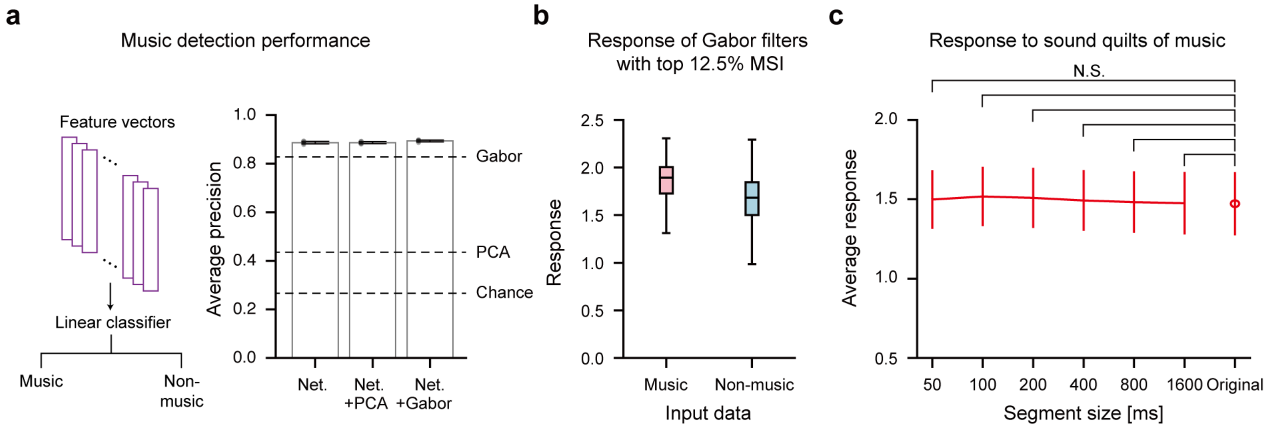

Supplementary Fig. 4. Analysis of feature vectors obtained by linear methods.

(**a**) Binary classification of the data using a linear regression classifier. Baselines (linear models, chance) are indicated as black dotted lines. Error bars represent the standard deviation for different network initialization conditions. (**b**) Response of the Gabor filters with top 12.5% MSI to music (red) and non-music (blue) stimuli. The box represents the lower and upper quartile. The whiskers represent the lower (upper) quartile – (+) 1.5 × interquartile range. n_{music} = 4,539, $n_{non\text{-}music}$ = 10,483 sounds. (**c**) Response of the Gabor filters with top 12.5% MSI to sound quilts made of music. One-tailed Wilcoxon rank-sum test. U_{50} = 9,623,755, U_{100} = 8,755,292, U_{200} = 8,956,076, U_{400} = 9,580,856, U_{800} = 9,967,139, $U_{1,600}$ = 10,208,603; p_{50} = 1.000, p_{100} = 1.000, p_{200} = 1.000, p_{400} = 1.000, p_{800} = 0.996, $p_{1,600}$ = 0.771; ES_{50} = 0.467, ES_{100} = 0.425, ES_{200} = 0.435, ES_{400} = 0.465, ES_{800} = 0.484, $ES_{1,600}$ = 0.496. N.S.: non-significant. n = 4,539 sounds. Error bars represent mean +/- SD. Source data are provided as a Source Data file.

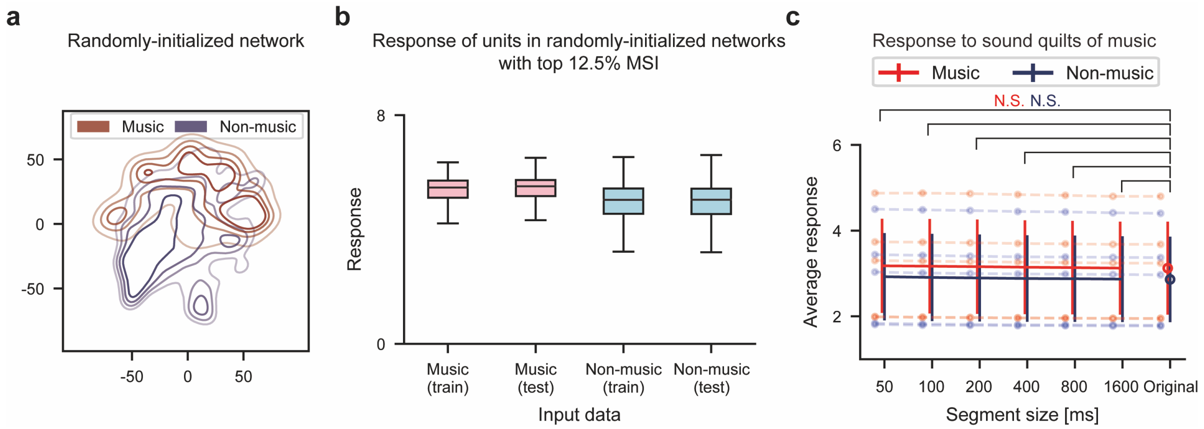

Supplementary Fig. 5. Analysis of the feature vectors of randomly-initialized networks.

(**a**) Density plot of the t-SNE embedding of feature vectors obtained from a randomly-initialized network. (**b**) Response of the units with top 12.5% MSI in the random network to music and non-music stimuli. The whiskers represent the lower (upper) quartile – (+) 1.5 × interquartile range. $n_{music, train}$ = 4,539, $n_{music, test}$ = 3,999, $n_{non-music, train}$ = 10,483, and $n_{non-music\_test}$ = 11,010 independent sounds. (**c**) Response of the units with top 12.5% MSI to sound quilts made of music (red) and non-music (blue). One-tailed Wilcoxon signed-rank test. For music quits: U_{50} = 0, U_{100} = 0, U_{200} = 0, U_{400} = 0, U_{800} = 0, $U_{1,600}$ = 5; p_{50} = 1.000, p_{100} = 1.000, p_{200} = 1.000, p_{400} = 1.000, p_{800} = 1.000, $p_{1,600}$ = 0.781; ES_{50} = 0, ES_{100} = 0, ES_{200} = 0, ES_{400} = 0, ES_{800} = 0, $ES_{1,600}$ = 0; For non-music quilts: U_{50} = 0, U_{100} = 0, U_{200} = 0, U_{400} = 0, U_{800} = 0, $U_{1,600}$ = 0; p_{50} = 1.000, p_{100} = 1.000, p_{200} = 1.000, p_{400} = 1.000, p_{800} = 1.000, $p_{1,600}$ = 1.000; ES_{50} = 0, ES_{100} = 0, ES_{200} = 0, ES_{400} = 0, ES_{800} = 0, $ES_{1,600}$ = 0; N.S.: non-significant. Error bars represent mean +/- SD. n = 5 independent networks. Source data are provided as a Source Data file.

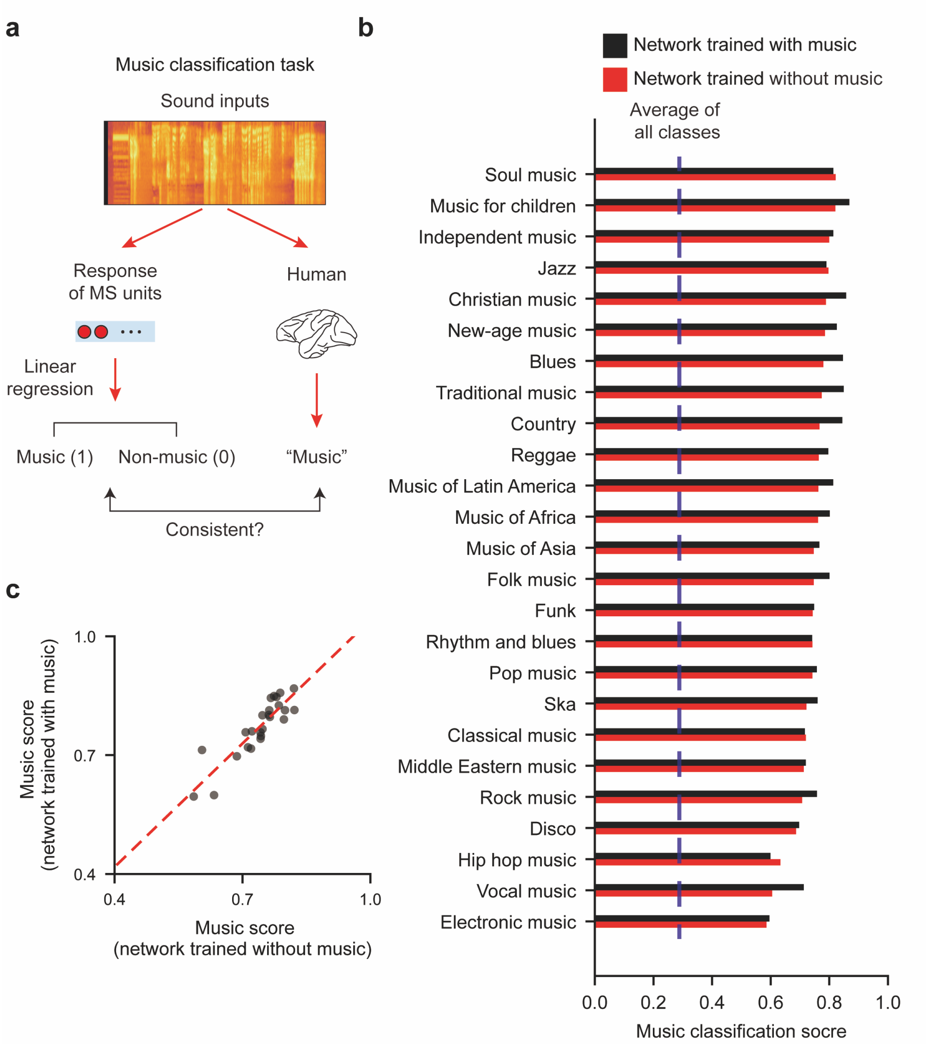

Supplementary Fig. 6. Music classification task using the music-selective units.

(**a**) Illustration of the music classification task using the music-selective units. (**b**) Music classification performance for all 25 music genres included in the dataset. The x-axis (music score) represents the average value of the output of the linear regression model (music = 1, non-music = 0) for each musical genre. $n_{Electronic} = 35$, $n_{Vocal} = 58$, $n_{Hiphop} = 38$, $n_{Disco} = 41$, $n_{Rock} = 57$, $n_{Middle\ Eastern} = 44$, $n_{Classical} = 47$, $n_{Ska} = 48$, $n_{Pop} = 50$, $n_{R\&B} = 37$, $n_{Funk} = 51$, $n_{Folk} = 46$, $n_{Asia} = 40$, $n_{Africa} = 60$, $n_{Latin\ America} = 42$, $n_{Reggae} = 43$, $n_{Country} = 58$, $n_{Traditional} = 54$, $n_{Blues} = 47$, $n_{New\text{-}age} = 44$, $n_{Christian} = 38$, $n_{Jazz} = 53$, $n_{Independent} = 50$, $n_{Children} = 18$, $n_{Soul} = 49$ independent sounds were investigated for 5 independent networks. (**c**) The classification score for the network trained with music is strongly correlated with that of the network trained without music for the 25 genres in (b) (Pearson's r = 0.884, $p = 4.655 \times 10^{-9}$). The dashed red line is a linear fit. Source data are provided as a Source Data file.

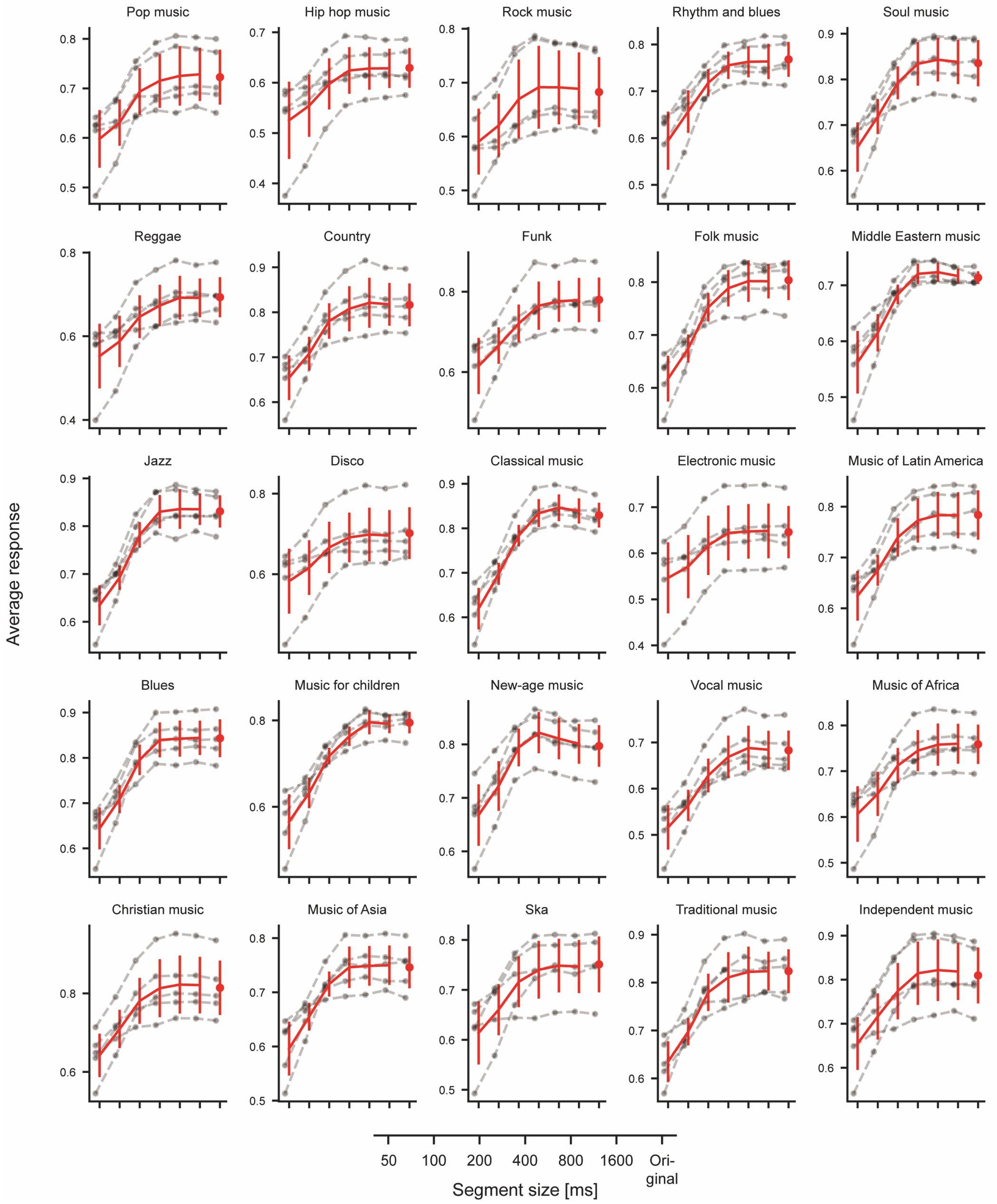

Supplementary Fig. 7. Analysis of response of the units in networks trained without music to sound quilts of different music genres.

Error bars represent mean +/- SD. n = 5 independent networks. Source data are provided as a Source Data file.

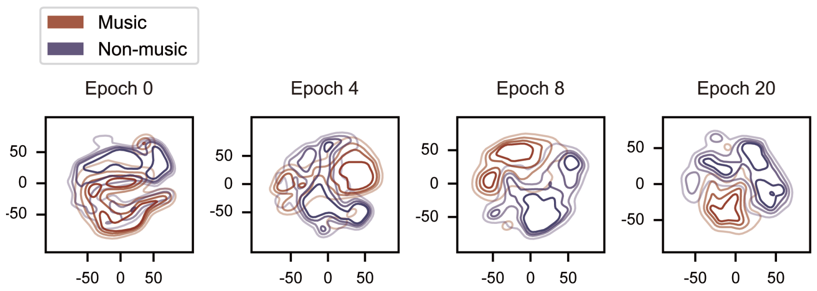

Supplementary Fig. 8. Density plots of the t-SNE embeddings of music (red) and non-music (blue) over the training epochs.

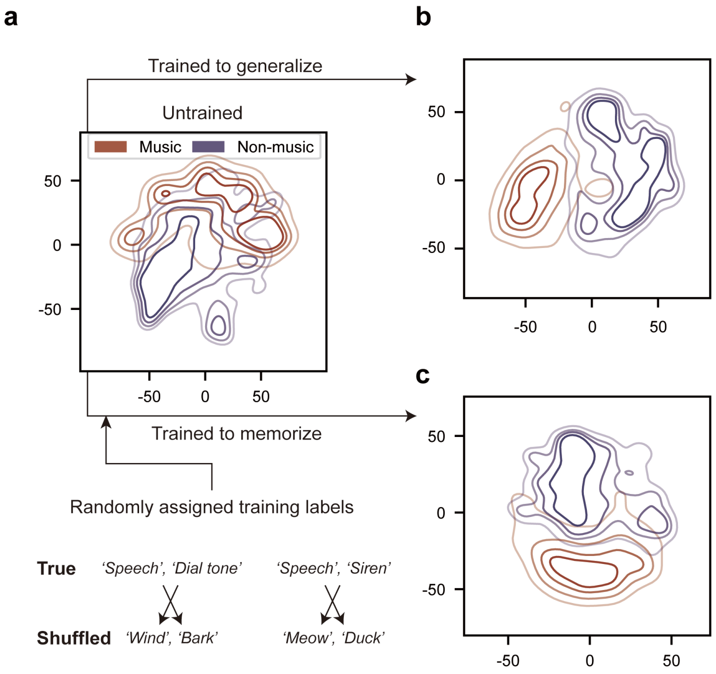

Supplementary Fig. 9. T-SNE embedding of the feature vectors of the network trained to memorize natural sounds with randomized labels.

(**a**) Illustration of network training to memorize the data by randomizing the labels. (**b**) Density plot of the t-SNE embedding of the feature vectors obtained from the network trained with the original label and (**c**) with the randomized labels.

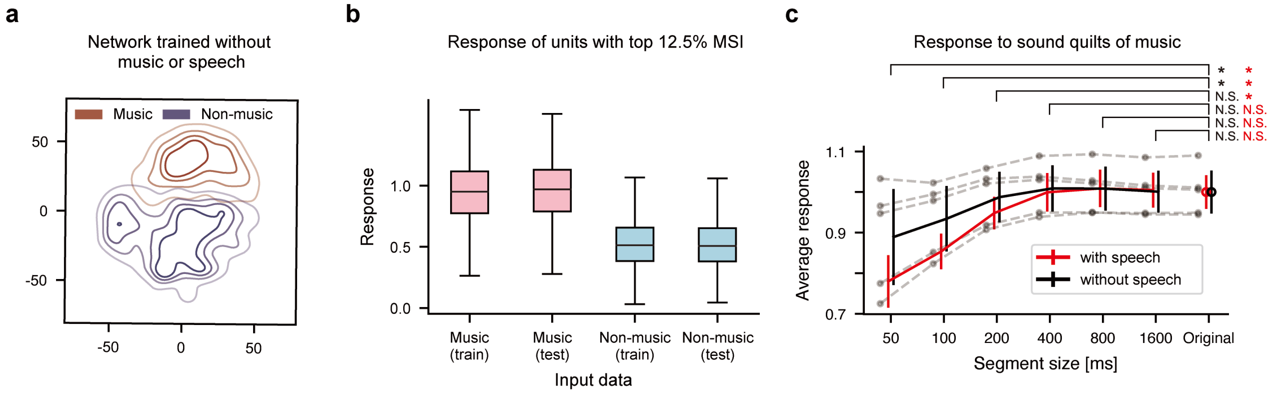

Supplementary Fig. 10. Analysis of the feature vectors of networks trained without music or speech. (**a**) Density plot of the t-SNE embedding of feature vectors obtained from a network trained without music or speech. (**b**) Response of the units with top 12.5% MSI in the network to music and non-music stimuli. The whiskers represent the lower (upper) quartile – (+) 1.5 × interquartile range. $n_{music, train}$ = 4,539, $n_{music, test}$ = 3,999, $n_{non\text{-}music, train}$ = 10,483, and $n_{non\text{-}music\_test}$ = 11,010 independent sounds. (**c**) Response of the units with top 12.5% MSI to sound quilts made of music. One-tailed Wilcoxon signed-rank test, U_{50} = 15, U_{100} = 15, U_{200} = 12, U_{400} = 4, U_{800} = 0, $U_{1,600}$ = 6; p_{50} = 0.031, p_{100} = 0.031, p_{200} = 0.156, p_{400} = 0.844, p_{800} = 1.000, $p_{1,600}$ = 0.688; ES_{50} = 1, ES_{100} = 1, ES_{200} = 0.8, ES_{400} = 0.267, ES_{800} = 0, $ES_{1,600}$ = 0.4; n = 5 independent networks. Error bars represent mean +/- SD. The asterisks indicate statistical significance ($p < 0.05$). N.S.: non-significant. Source data are provided as a Source Data file.

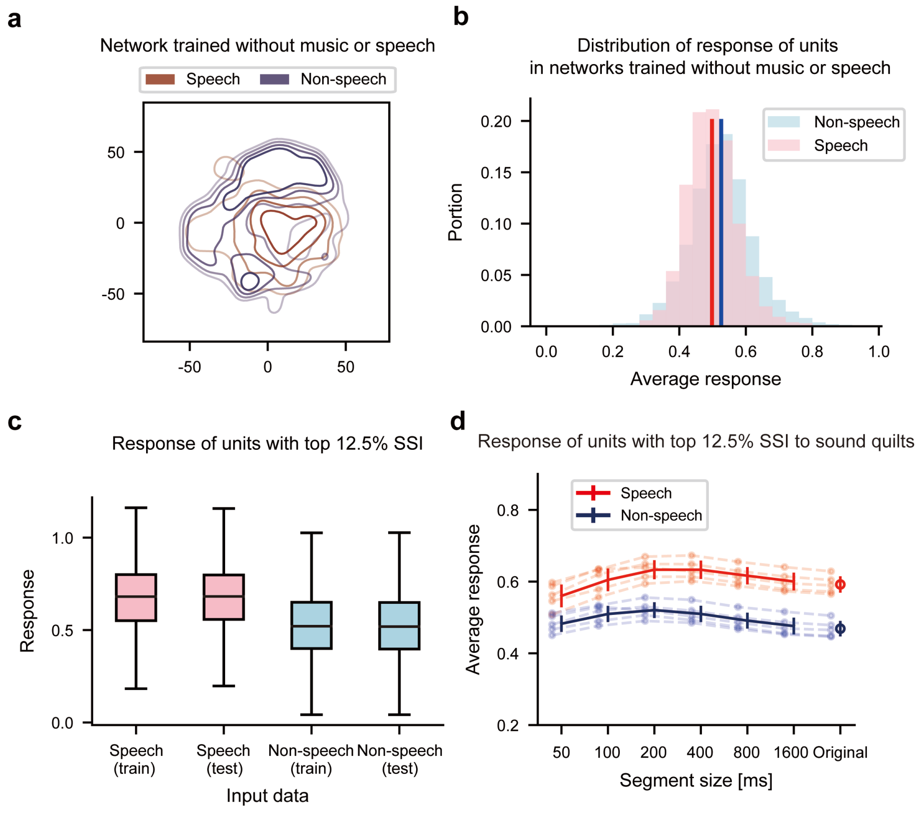

Supplementary Fig. 11. Representation of speech in networks trained without music or speech.

(**a**) Density plot of the t-SNE embedding of feature vectors obtained from a network trained without music or speech. (**b**) Histograms of the average response of the units for speech (red) and non-speech (blue) stimuli in networks trained without music or speech. The lines represent the response averaged over all units. (**c**) Response of the units with top 12.5% speech-selectivity index (SSI) in the network to speech and non-speech stimuli (all inputs with normalized amplitude). The whiskers represent the lower (upper) quartile – (+) 1.5 × interquartile range. (**d**) Response of the units with top 12.5% SSI to sound quilts made of speech (red) and non-speech (blue). n = 5 independent networks. Error bars represent mean +/- SD. Source data are provided as a Source Data file.

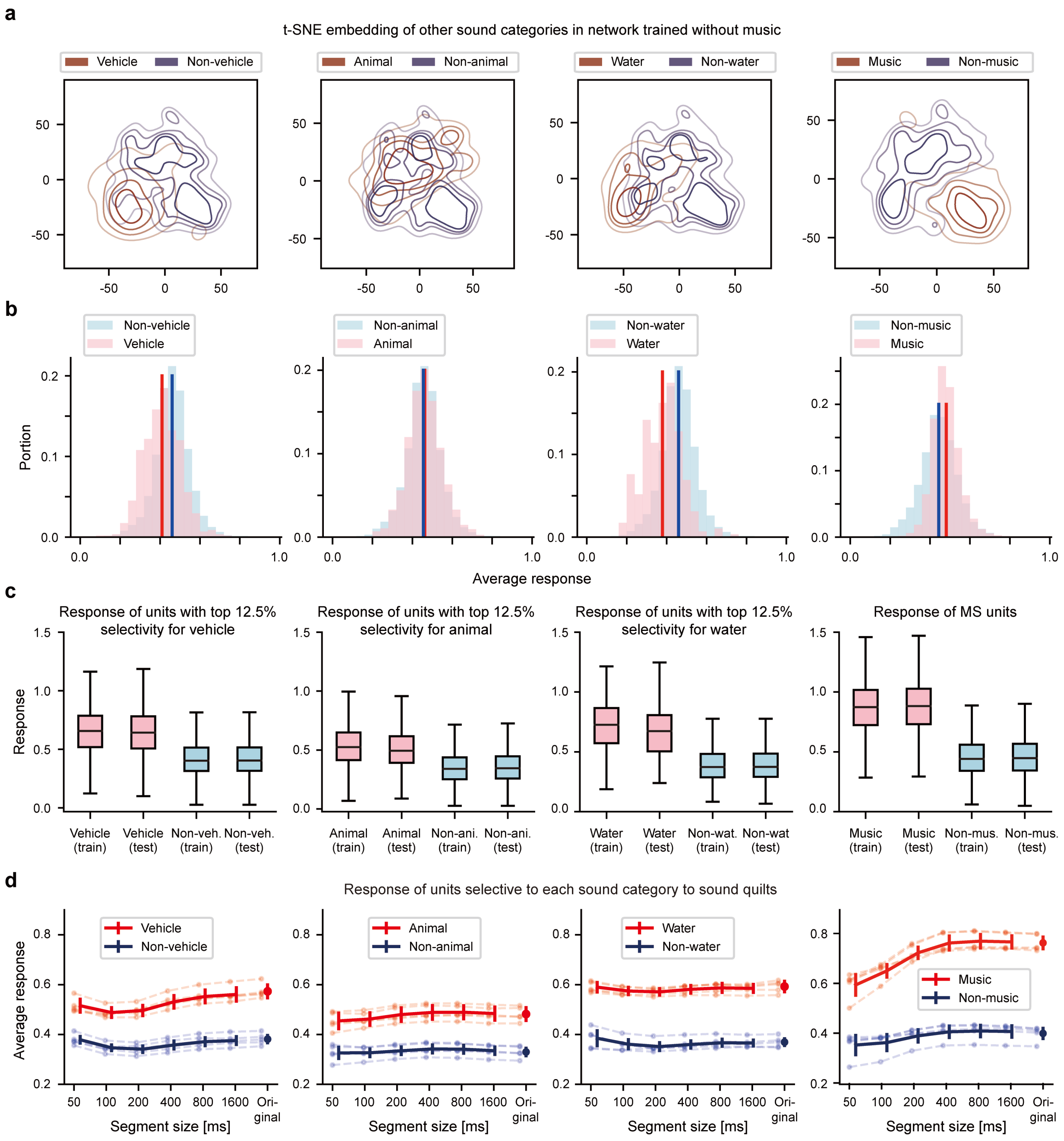

Supplementary Fig. 12. Analysis of the encoding of other sound categories.

(**a**) Density plot of the t-SNE embedding of feature vectors of various sound inputs (RMS normalized) obtained from the network trained without music. As in the analysis of music-selectivity, vehicle-related categories were defined as all classes under the vehicle hierarchy, and sound data containing only vehicle-related categories ('vehicle') or containing no vehicle-related categories ('non-vehicle') were used in the selectivity analysis (the same applies to animal and water). (**b**) Histograms of the average response of the units for different sound categories in networks trained without music. (**c**) Units with the top 12.5% class

selectivity values show stronger response to the specific class than other sounds in the training dataset on average (1.46, 1.45, and 1.63 times for vehicle, animal, and water respectively). The whiskers represent the lower (upper) quartile – (+) 1.5 × interquartile range. $n_{music,\ train}$ = 4,539, $n_{music,\ test}$ = 3,999, $n_{non\text{-}music,\ train}$ = 10,483, and $n_{non\text{-}music\_test}$ = 11,010 independent sounds. (**d**) Response of the units with top 12.5% class selectivity values (from left, vehicle, animal, and water respectively) to sound quilts. n = 5 independent networks. Error bars represent mean +/- SD. Source data are provided as a Source Data file.

Layer	Type	Output Shape	Kernels	Activations
Input	Log-Mel spectrogram input	64 × 802 x 1<br>(height x width x channel)		
Conv1	Convolution	30 × 200 x 32	Size: 5 × 5 × 1 × 32<br>Stride: 2 x 4	Batch normalization and ReLU
Pool1	Max pooling	15 × 100 x 32	Size = 2 x 2<br>Stride = 2	Dropout (p = 0.2)
Conv2	Convolution	11× 96 x 64	Size: 5 × 5 × 32 × 64<br>Stride: 1	Batch normalization and ReLU
Pool2	Max pooling	10 × 95 x 64	Size = 2 x 2<br>Stride = 1	
Conv3	Convolution	6 × 91 x 128	Size: 5 × 5 × 64 × 128<br>Stride = 1	Batch normalization and ReLU
Pool3	Max pooling	5 × 90 x 128	Size: 2 x 2<br>Stride: 1	Dropout
Conv4	Convolution	1 × 86 x 256	Size: 5 × 5 × 192 × 256<br>Stride: 1	Batch normalization, ReLU, and dropout
AvgPool1	Global average pooling	1 × 1 x 256		
FC1	Fully Connected	256	Weights: 256 × 256<br>Bias: 256 × 1	ReLU and dropout
FC2 (Output)	Classification Output	527	Weights: 527 × 256<br>Bias: 527 × 1	Sigmoid

Supplementary Table 1. Summary of the network architecture.

The network consists of four convolutional layers for feature extraction (Conv1 – Conv4) and two fully connected layers for natural sound detection (FC1 – FC2). We note that the specific choice of hyperparameters does not significantly change the results in the main text.
